# Mechanisms of synergistic antibacterial and anti-biofilm effects of dihydroquercetin combined with ceftazidime against mucoid *pseudomonas aeruginosa*

**DOI:** 10.3389/fmicb.2026.1945324

**Published:** 2026-09-09

**Authors:** Li Gao, Wenjun Lu, Keda Chen, Xuedan Qiu, Qingcao Li

**Affiliations:** 1 Department of Blood Transfusion, The Affiliated Lihuili Hospital of Ningbo University, Ningbo, China; 2 Department of Intensive Care Unit, The Affiliated Lihuili Hospital of Ningbo University, Ningbo, China; 3 Department of Clinical Laboratory, The Affiliated Lihuili Hospital of Ningbo University, Ningbo, China

**Keywords:** biofilm, ceftazidime, combined drug sensitivity, dihydroquercetin, mucoid *Pseudomonas aeruginosa*

## Abstract

**Objective:**

This study characterized the synergistic activity of dihydroquercetin combined with ceftazidime (CAZ) against mucoid *Pseudomonas aeruginosa* (mPA). The regimen primarily relieves biofilm-mediated phenotypic tolerance in CAZ-susceptible isolates, rather than reversing classical genetic resistance, while also enhancing efficacy against a minor proportion of intermediate/resistant strains. We further clarified its mechanisms via membrane integrity tests, qRT-PCR of biofilm/quorum-sensing genes and untargeted metabolomics, to provide experimental basis for treating refractory biofilm-related mPA infections.

**Methods:**

Forty-one clinical mPA isolates were screened via the K-B assay. Checkerboard FICI and time-kill assays evaluated combined antibacterial effects Mucoid EPS semi‑quantitative staining assessed mucoid phenotype. Biofilm biomass and membrane permeability were measured by crystal violet staining and ALP leakage. qRT-PCR quantified biofilm/quorum-sensing gene expression. UHPLC-Orbitrap untargeted metabolomics coupled with multivariate analysis identified differential metabolites. Statistics were analyzed using GraphPad Prism and the R package ropls.

**Results:**

87.8% of the 41 mPA isolates were ceftazidime-susceptible. Mucoid EPS semi‑quantitative staining showed dihydroquercetin‑ceftazidime co‑treatment markedly repressed mucoid EPS, particularly in resistant mPA isolates. Over 90% of strains displayed synergistic (31.7%, FICI ≤0.5) or additive (61.0%, 0.5 < FICI ≤1.0) responses to the combination. Time-kill curves showed the combination achieved a ≥ 2 log₁₀ CFU/mL reduction and suppressed early bacterial growth at 4–8 h. Single drugs weakly inhibited biofilms, whereas co-treatment sharply lowered biofilm biomass (*p* < 0.01). Dihydroquercetin increased membrane permeability in a time-dependent manner, with stronger effects in the combination group. qRT-PCR revealed co-treatment significantly downregulated lasR, rhlR, pslA, pelA and fliC (*p* < 0.05). Metabolomics confirmed the combination triggered broader metabolic reprogramming, reducing virulence metabolites and biofilm precursors while elevating citric acid, a core TCA intermediate. KEGG enrichment highlighted perturbed valine/leucine/isoleucine biosynthesis, pyruvate metabolism and purine metabolism.

**Conclusion:**

Dihydroquercetin synergizes with CAZ against mPA by disrupting biofilm/membrane integrity, repressing quorum-sensing and biofilm-related genes, and remodeling bacterial metabolism. This combination can treat biofilm-associated mPA infections and validates dihydroquercetin as a plant-derived antibiotic adjuvant.

## 1 Introduction

Mucoid *Pseudomonas aeruginosa* (mPA) causes intractable chronic infections, including cystic fibrosis-associated pneumonia (Pier, 2002) and burn wound infections (Liao et al., 2022). Biofilm formation generates an extracellular polymeric substance (EPS) matrix that occupies 85% of the total biofilm volume (Jamal et al., 2018), conferring strong antibiotic tolerance and frequently leading to treatment failure (Høiby et al., 2010; Venkatesan et al., 2015; Yin et al., 2019). The EPS matrix hinders penetration of ceftazidime, azithromycin and imipenem, enabling persistent survival of embedded sessile bacteria (Kurz et al., 2021; Liu K. et al., 2022).

Dihydroquercetin (taxifolin, chemical name: 3,3′,4′,5,7-pentahydroxyflavanone), a natural plant-derived flavonoid, possesses multiple pharmacological activities, including antioxidant (Kuang et al., 2017), anti-tumor (Chen et al., 2018), antibacterial (Sunil and Xu, 2019), and anti-biofilm properties (Pal and Tripathi, 2019), qualifying it as a promising plant-derived bacteriostatic adjuvant. Unlike synthetic antibiotics, plant-derived antimicrobials rarely induce resistance and represent promising adjuvants for infection control (Sharma et al., 2018).

Clinically, most planktonic mPA isolates are susceptible to ceftazidime (Bonfiglio and Marchetti, 2000; Fleming and Knie, 1981), yet biofilms cause non-genetic phenotypic tolerance that weakens ceftazidime monotherapy. This study thus targets biofilm-induced tolerance in susceptible strains instead of reversing genetic resistance. Single agents have limited effects, and their synergism against both susceptible and resistant/intermediate mPA remains unreported. We assessed their *in vitro* combination to treat biofilm-related mPA infections.

## 2 Materials and methods

### 2.1 Isolate collection and identification

Forty-one non-repetitive clinical mPA isolates were recovered from multiple specimens (32 isolates from sputum and throat swabs, 7 from bronchoalveolar lavage fluid (BALF), and 2 from other specimen types) collected in Ningbo between January 2020 and June 2023. Basic clinical information for all isolates is provided in Supplementary Table 1. All isolates were first identified as *P. aeruginosa* via MALDI-TOF MS (Zhongyuan, China). Strains forming convex, glossy, jelly-like colonies that produced visible mucus strands on blood agar were defined as putative mucoid isolates. The mucoid phenotype was further verified by the modified Muir method (Owlia et al., 2014). Eric-PCR was applied for genotypic confirmation and strain discrimination to exclude clonal duplicates. The study was approved by the Ethics Committee of Lihuili Hospital, Ningbo University (KY 2023 SL 328-01).

### 2.2 Reagents and materials

Dihydroquercetin (GLP Bioscience), ceftazidime (Meilunbio), Dimethyl sulfoxide (DMSO, Sangon Biotech); 30 μg ceftazidime K-B susceptibility discs (OXOID, United Kingdom); broth dilution kits (Wenzhou Kangtai); Columbia blood agar and MH agar plates (Autobio); Alkaline Phosphatase Assay Kit (Beyotime); RNA extraction, cDNA synthesis, and qPCR kits (BGI, Shenzhen); HPLC-grade methanol, acetonitrile, formic acid, water, and acetone (Fisher Scientific).

### 2.3 Drug preparation

Dihydroquercetin was dissolved in DMSO and sterile-filtered through a 0.22 μm membrane to prepare a 4,096 mg/L stock solution. The stock solution was stored in a brown glass bottles under dark refrigeration. Appropriate dilutions of the stock solution were prepared prior to addition into bacterial cultures. The final concentration of DMSO was maintained ≤ 0.05% (v/v) across all treatment groups. As reported in existing literature, DMSO at such a low concentration exerts negligible impacts on the growth and biofilm formation of *Pseudomonas aeruginosa* (Kirkwood et al., 2018).

### 2.4 Instruments and equipment

Multimode microplate reader (Molecular Devices); shaking incubator; constant incubator; ABI 7500 qPCR system (Roche); UHPLC-Orbitrap Exploris 240 mass spectrometer (Thermo Fisher); HSS T3 column (100 mm × 2.1 mm i.d., 1.8 μm, Waters).

### 2.5 Combined antibacterial activity of dihydroquercetin and ceftazidime

#### 2.5.1 Selection of experimental strains

Mueller–Hinton (MH) agar plates inoculated with standardized bacterial suspensions were subjected to the Kirby–Bauer disk diffusion assay using commercial ceftazidime discs. Following incubation, inhibition zone diameters were measured to categorize isolate susceptibility. One susceptible, one intermediate and one resistant mPA isolate were selected to perform all subsequent phenotypic and mechanistic experiments, including Mucoid EPS Semi-quantitative Staining Assay, time-kill curve assays, biofilm quantification, membrane permeability detection, qRT-PCR, and untargeted metabolomics.

#### 2.5.2 MIC determination of dihydroquercetin and ceftazidime

Minimum inhibitory concentrations (MICs) were determined via broth microdilution. Single mPA colonies were shaken in CAMHB at 37 °C for 24 h, and suspensions were adjusted to 1 × 10^6^ CFU/mL (100 μL per well in 96-well plates). Dihydroquercetin and ceftazidime (dissolved in sterile water) were two-fold serially diluted in CAMHB (2048–4 μg/mL and 256–0.125 μg/mL, respectively). Each well contained 100 μL of bacteria plus 100 μL drug solution (total 200 μL). Sterile CAMHB and untreated bacterial culture served as negative and positive controls. After 24 h incubation at 37 °C, MIC was defined as the lowest concentration with no visible bacterial growth.

#### 2.5.3 Determination of fractional inhibitory concentration index

Checkerboard microdilution assays were performed to calculate the fractional inhibitory concentration index (FICI). Bacterial suspensions and drug solutions were diluted as described in Section 2.5.2. Briefly, 50 μL serially diluted dihydroquercetin and ceftazidime were dispensed into 96-well plates in a checkerboard layout, followed by the addition of 100 μL standardized bacterial suspension. Negative and positive controls were included consistently with Section 2.5.2, and plates were incubated at 37 °C for 24 h. The FICI was calculated using the formula: FICI = (MIC_A_ combination/MIC_A_ alone) + (MIC_B_ combination/MIC_B_ alone).

Combinatorial antibacterial interactions were classified according to previously published criteria: synergistic activity (FICI ≤0.5), additive effect (0.5 < FICI ≤1), indifferent effect (1 < FICI ≤2), and antagonistic effect (FICI >2) (Ma et al., 2024).

### 2.6 Mucoid EPS semi-quantitative staining assay

Briefly, isolate suspensions were thinly smeared, air-dried, covered with filter paper saturated in Ziehl–Neelsen carbon ink, and steamed for 30 s. Slides were rinsed sequentially with 95% ethanol and distilled water, treated with mordant for 20 s, washed again, and decolorized with ethanol. Samples were counterstained in 0.3% methylene blue for 30–60 s and observed under oil immersion microscopy; bacterial cytoplasm stained red and mucoid EPS matrix stained blue (Owlia et al., 2014).

Mucoid EPS was semi-quantified by measuring blue halo width around red cells at 100 × magnification (10 random fields, 3 technical replicates per isolate): non-mucoid (−): No visible blue halo surrounding bacterial cells; very weak (+): Thin blue capsular halo ≤1 μm; weak-moderate (++): Halo 1–3 μm; moderate (+++): Halo 3–6 μm; strong (++++): Halo >6 μm.

### 2.7 Time-kill curve assay

Time-kill assays were conducted to evaluate the bactericidal activity of dihydroquercetin combined with CAZ at 0.5 × combined MIC. Standardized mPA suspensions were incubated with single agents or the drug combination at 35 °C. Aliquots were sampled at 0, 2, 4, 6, 8, and 24 h, serially diluted, and plated for colony counting (triplicate tests). Killing profiles were plotted as log₁₀ CFU/mL versus time. Activity was categorized by log reduction thresholds (Rizvi et al., 2013): bactericidal (≥3-log CFU drop), synergism (≥2-log CFU drop), indifference (<2-log CFU change), and antagonism (>2-log increase).

### 2.8 Biofilm quantification

Biofilm biomass was quantified via crystal violet staining. Overnight mPA cultures were diluted 1:100 in LB medium (200 μL per well), treated with single agents or the combination at 0.5 × combined MIC, and incubated statically at 37 °C for 48 h. Wells were washed twice with saline to remove planktonic bacteria, air-dried, fixed with methanol for 15 min, stained with crystal violet for 15 min, rinsed, and air-dried again. The bound crystal violet was dissolved in absolute ethanol, and absorbance was measured at 595 nm.

### 2.9 Membrane permeability assay

Bacterial suspensions (1 × 10^6^ CFU/mL) were cultured in LB medium containing 0.5 × combined MIC dihydroquercetin, ceftazidime, their combination, or drug-free vehicle control at 37 °C with shaking for 24 h. After centrifugation (5,000 rpm, 5 min), supernatants were harvested to quantify extracellular alkaline phosphatase (ALP) activity; ALP leakage serves as an indicator of compromised membrane integrity. ALP activity was colorimetrically detected at 420 nm using p-nitrophenyl phosphate (pNPP) as the substrate, which develops a yellow chromophore upon dephosphorylation.

### 2.10 qRT-PCR analysis of biofilm-related genes

qRT-PCR was performed to quantify the transcription levels of biofilm-associated genes (*lasR, rhlR, pslA, pelA, fliC*) using *oprL* as the internal reference; these genes are involved in quorum sensing, exopolysaccharide synthesis and flagellar motility. Primers for *fliC*, *pslA* and *pelA* were newly designed using Primer Premier 6.0 based on the reference sequences of PA deposited in the National Center for Biotechnology Information (NCBI). In contrast, the primer sequences of *oprL*, *lasR* and *rhlR* were retrieved from published literature (Joly et al., 2005; Chan et al., 2015). All primer pairs listed in Table 1 were commercially synthesized by BGI Genomics (Shenzhen, China). Total RNA was extracted from mPA cultured for 16–18 h with 0.5 × combined MIC dihydroquercetin, CAZ, their combination or drug-free LB medium, RNA purity and concentration were detected by NanoDrop, and RNA was reverse-transcribed into complementary DNA (cDNA). The 2^−^ΔΔCt method was adopted to calculate relative gene expression (Ren et al., 2024).

**Table 1:** The primers used in this study.

Gene	Primer sequence	Amplicon size (bp)	References
oprL	5’-AACAGCGGTGCCGTTGAC-3′	87	Joly et al. (2005)
	5’-GTCGGAGCTGTCGTACTCGAA-3′		
lasR	5’-CTGTGGATGCTCAAGGACTAC-3′	133	Chan et al. (2015)
	5’-AACTGGTCTTGCCGATGG-3′		
fliC	5’-CGACAAGGGTGTACTGACCA-3′	100	
	5’-GACCTTCACTGCGACCTGAC-3′		
pslA	5’-CGGTCAGCGAATACAGCTC-3′	103	
	5’-TTGATCTTGTGCAGGGTGTC-3′		
pelA	5’-GGAACAGCCAGGTAATGGAC-3′	112	
	5’-TCCAGGGTATCGAGGAACAG-3′		
rhlR	5’-GCCAGCGTCTTGTTCGG-3’	160	Chan et al. (2015)
	5’-CGGTCTGCCTGAGCCATC-3′		

### 2.11 Untargeted metabolomics via LC–MS

#### 2.11.1 Grouping setup

Four groups were established: DX1 (dihydroquercetin monotherapy), DX2 (ceftazidime monotherapy), DX3 (dihydroquercetin plus ceftazidime combination), and DX4 (blank control). Single and combined treatment groups received drugs at 0.5 × combined MIC, while the control group received only solvent vehicle.

#### 2.11.2 Sample pretreatment

Briefly, 50 ± 5 mg bacterial cell pellets with grinding beads were mixed with 400 μL extraction solvent (methanol:water = 4:1, v/v) spiked with four internal standards including L-2-chlorophenylalanine (0.02 mg/mL). Samples were subjected to cryogenic grinding, sonication and cold incubation, followed by centrifugation at 13,000 × g (4 °C, 15 min). Supernatants were collected for LC–MS analysis. Equal volumes of all individual supernatants were pooled to generate quality control (QC) samples.

#### 2.11.3 LC–MS detection

Metabolite profiling was conducted on a UHPLC-Orbitrap Exploris 240 mass spectrometer equipped with an HSS T3 analytical column. Mobile phases A and B contained formic acid, and gradient elution was performed at 40 °C with a flow rate of 0.40 mL/min. The mass spectrometer operated in dual positive and negative ion modes with an m/z scan range of 70–1,050. Spray voltage, gas parameters and stepped collision energies were optimized for consistent metabolite acquisition.

### 2.12 Statistical analysis

GraphPad Prism 10.2 software was used for graphical plotting. Two-group comparisons were performed using the Mann–Whitney U test; multi-group comparisons adopted the Kruskal–Wallis H test with Dunn’s correction. *p* < 0.05 indicated statistical significance. PCA and OPLS-DA were conducted via the R package ropls. Analysis of similarities (ANOSIM), a nonparametric permutation test, was further applied to assess the significance of intergroup metabolic separation. ANOSIM was calculated based on Bray-Curtis distance with 999 permutations using the anosim function in the R package vegan. Metabolites with VIP > 1 and *p* < 0.05 were defined as differential metabolites.

## 3 Results

### 3.1 Susceptibility of mPA to ceftazidime

**Figure 1:**
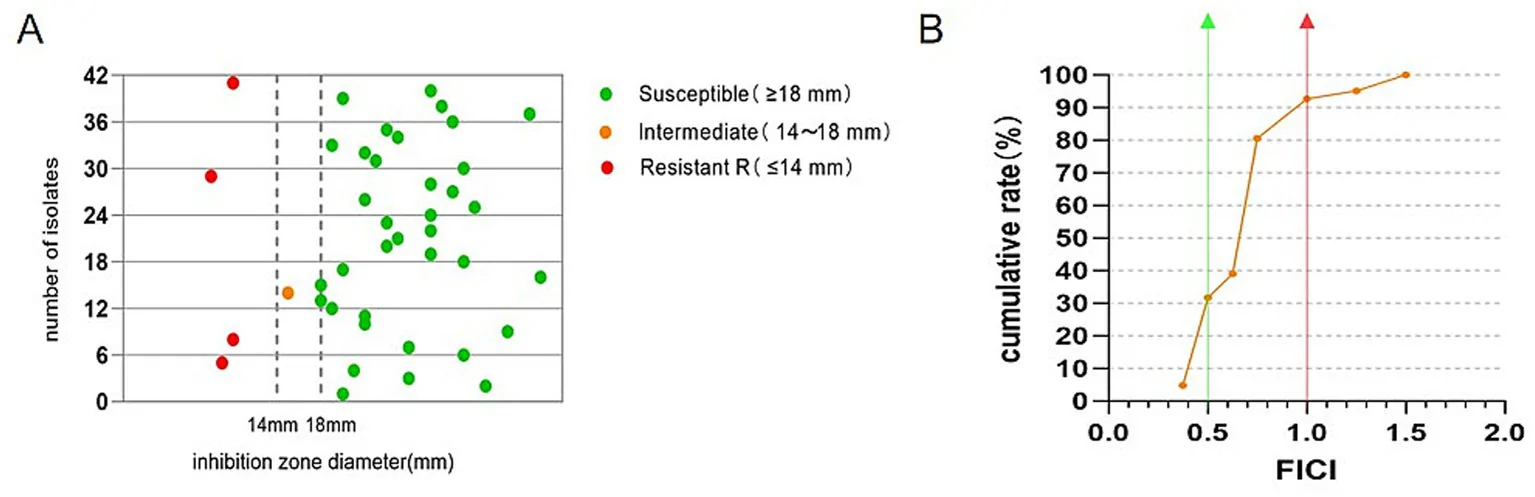
**(A)** Scatter plot of inhibition zone diameters of 41 mPA isolates against ceftazidime via K-B disk diffusion; **(B)** Distribution of fractional inhibitory concentration index (FICI) values for the combination of dihydroquercetin and ceftazidime against 41 mPA strains. Three technical replicates were set for each sample.

K-B disk diffusion results of 41 mPA strains are shown in Figure 1A, including 36 susceptible (87.8%), 1 intermediate (2.4%), and 4 resistant (9.8%) isolates. One strain from each susceptibility category was selected for follow-up experiments.

### 3.2 Combined antibacterial activity of dihydroquercetin and ceftazidime

Supplementary Figure S1 displays MIC values of single and combined agents. FICI distribution (Figure 1B) showed synergy (FICI ≤0.5) occurred in 31.7% strains, and additive effects (0.5 < FICI ≤1.0) in 61.0% strains, accounting for a total of 92.7%. Meanwhile, 7.3% of strains exhibited indifferent interactions (1 < FICI ≤2), while no antagonism observed (FICI >2). The combination exerted prominent synergistic or additive activity against most mPA.

### 3.3 Mucoid EPS semi-quantitative staining assay

Semi-quantitative capsule staining was used to evaluate mucoid EPS secretion in three mPA isolates (susceptible, intermediate, resistant) exposed to 0.5 × combined MIC of drugs at 0 h and 24 h (Table 2). Untreated controls exhibited steady EPS levels over 24 h. Each single drug partially inhibited EPS synthesis, whereas dihydroquercetin-ceftazidime co-treatment markedly reduced mucoid production in all isolates, especially the resistant strain, confirming the regimen efficiently alleviates the mucoid phenotype of mPA.

**Table 2:** Semi-quantitative detection of mucoid EPS secretion in three mPA isolates with distinct ceftazidime susceptibility profiles following drug incubation.

Isolate type	Time point	Dihydroquercetin monotherapy	Ceftazidime monotherapy	Combination medication	Control group
Susceptible (S)	0 h	**++**	**++**	**++**	**++**
	24 h	**+**	**++**	**+**	**+++**
Intermediate (I)	0 h	**+++**	**+++**	**+++**	**+++**
	24 h	**++**	**++**	**+**	**+++**
Resistant (R)	0 h	**++++**	**++++**	**++++**	**++++**
	24 h	**+++**	**++++**	**++**	**++++**

### 3.4 Time-kill curve analysis

**Figure 2:**
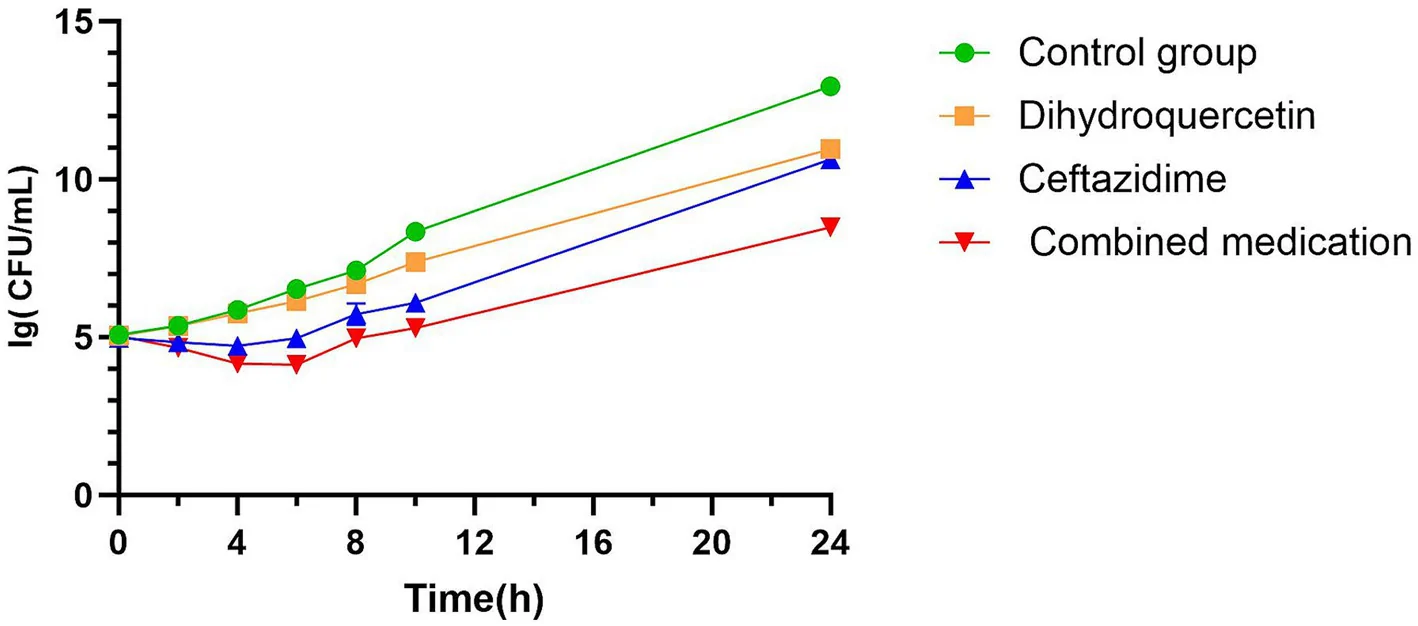
Time-kill curves of dihydroquercetin, ceftazidime, and their combination. Three biological replicates per group, with three technical replicates for each sample.

Figure 2 presents divergent growth profiles among groups. The untreated control rose from an initial density of 5.08 ± 0.67 log₁₀ CFU/mL to 12.94 ± 0.11 log₁₀ CFU/mL at 24 h, reflecting vigorous bacterial proliferation in the absence of drugs. Monotherapy with dihydroquercetin or ceftazidime only mildly retarded growth, with final counts reaching 10.95 ± 0.10 and 10.63 ± 0.27 log₁₀ CFU/mL, respectively.

By contrast, the combination group yielded 8.48 ± 0.08 log₁₀ CFU/mL at 24 h, corresponding to a 2.15 log₁₀ reduction relative to the most effective monotherapy group (ceftazidime alone), which meets the criterion for synergistic activity (≥2 log₁₀ CFU/mL drop). Marked early growth suppression was observed at 4–8 h in the combined treatment, confirming time-dependent synergistic inhibition.

### 3.5 Biofilm formation quantification

**Figure 3:**
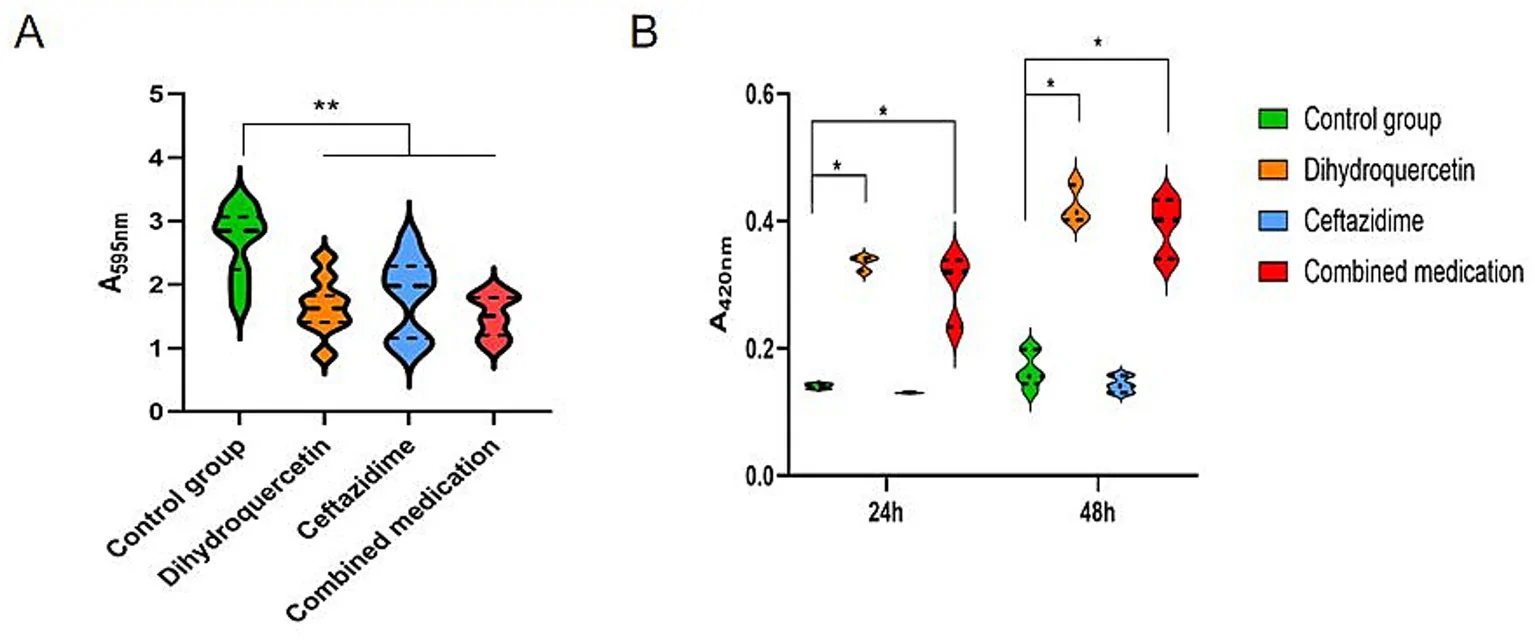
**(A)** Impacts of monotherapy and combination therapy on biofilm formation; **(B)** Effects of monotherapy and combination therapy on bacterial cell membrane permeability. *, *p* < 0.05; **, *p* < 0.01. Four-group comparisons: Kruskal-Wallis H test with Dunn’s post-hoc correction. Three biological replicates per group, with three technical replicates for each sample.

Biofilm biomass was quantified via crystal violet staining at A₅₉₅ (Figure 3A). The untreated control exhibited the highest absorbance, indicating abundant biofilm production. Single-agent treatment reduced biofilm biomass, whereas the combination produced markedly stronger inhibition (*p* < 0.01). Dihydroquercetin synergistically potentiated ceftazidime against biofilm formation.

### 3.6 Bacterial membrane permeability assay

Extracellular alkaline phosphatase (ALP) absorbance at 420 nm reflected membrane integrity (Figure 3B). At 24 and 48 h, A₄₂₀ values of dihydroquercetin alone and combined groups were significantly higher than the control (*p* < 0.05), while ceftazidime monotherapy showed no obvious difference versus control (*p* > 0.05). Dihydroquercetin alone or combined with ceftazidime increased membrane permeability in a time-dependent manner, with superior effects for the combination.

### 3.7 Expression of biofilm-related genes

**Figure 4:**
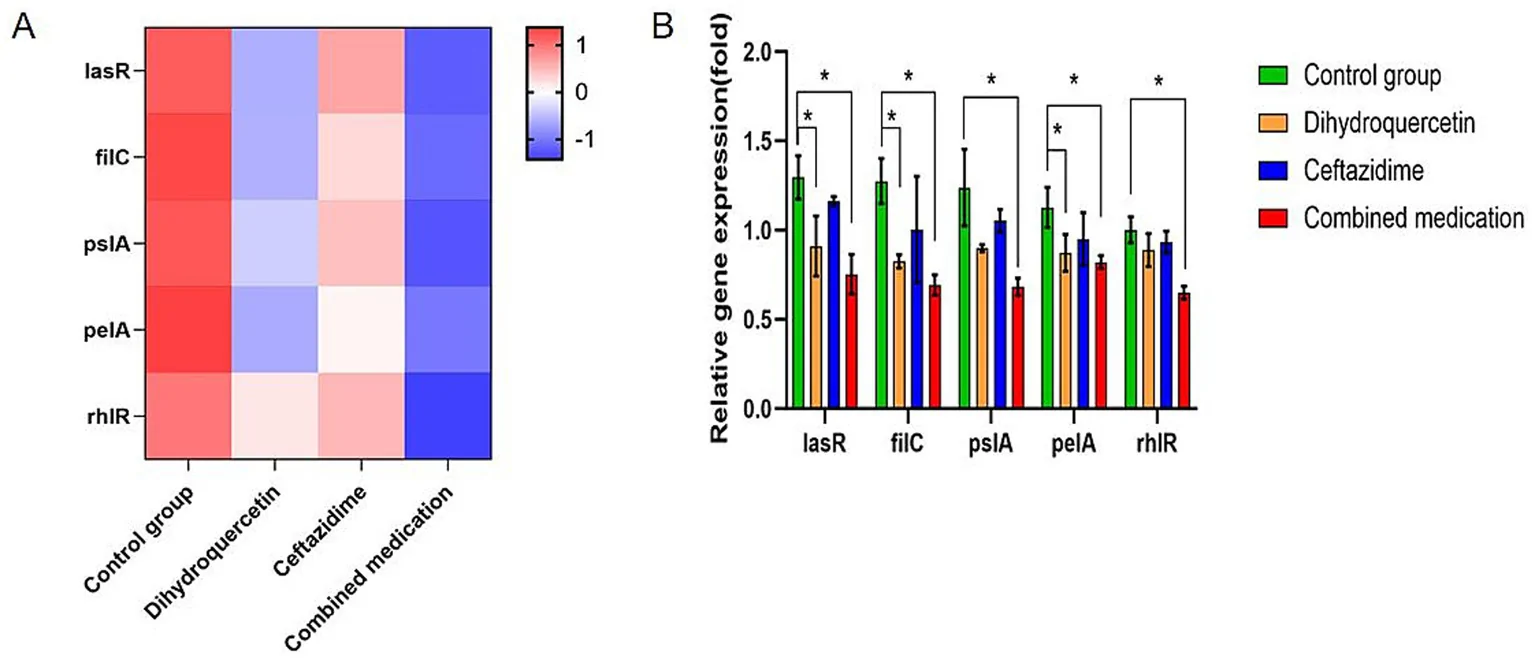
**(A)** Z-score-based expression heatmap of *lasR, fliC, pslA, pelA,* and *rhlR*; red = high expression, blue = low expression; **(B)** The expression levels of biofilm formation-related genes in Monotherapy and Combination Therapy. *, *p* < 0.05; **, *p* < 0.01. Four-group comparisons: Kruskal-Wallis H test with Dunn’s post-hoc correction. Three biological replicates per group, with three technical replicates for each sample.

qRT-PCR quantified transcription of five biofilm-associated genes (*lasR, fliC, pslA, pelA, rhlR*) (Figure 4), with consistent trends in heatmap and quantitative bar charts. All target genes were highly expressed in the untreated control. Monotherapy slightly suppressed gene transcription, while the combination markedly downregulated all five genes (*p* < 0.05 vs. control and single-agent groups). These data demonstrate that dihydroquercetin synergizes with ceftazidime to repress biofilm-related transcription and inhibit biofilm formation.

### 3.8 Metabolomic profiling

**Figure 5:**
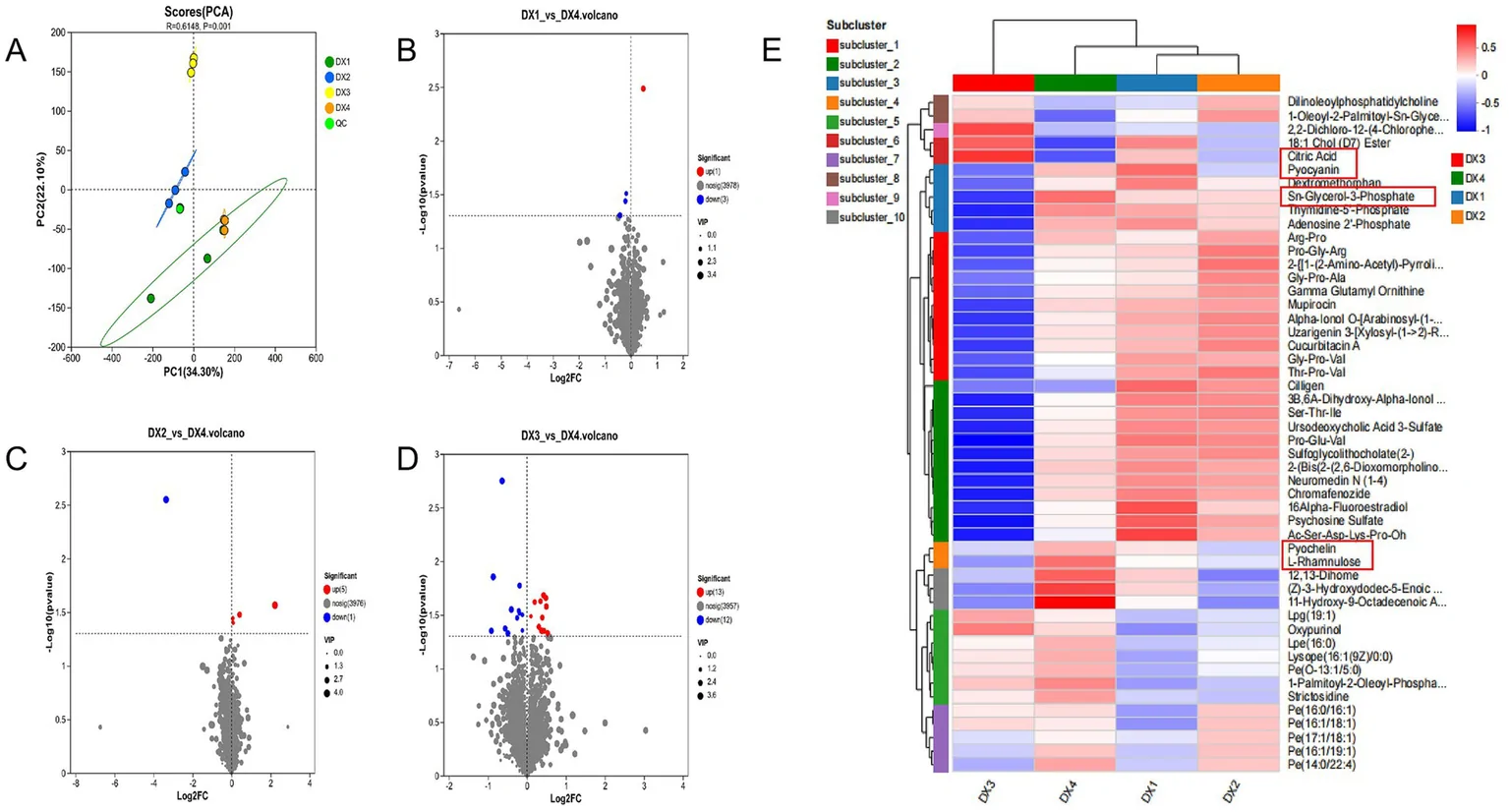
Multivariate metabolomic analysis of four experimental cohorts. **(A)** Principal component analysis (PCA) score plot including DX1, DX2, DX3, DX4, and QC pooled samples. The annotated *R* and *p* values were generated by Analysis of Similarities (ANOSIM) based on Bray–Curtis distance with 999 permutations. ANOSIM *R* ranges from −1 to 1; values close to 1 indicate far greater inter-group variation than intra-group variation, while values near 0 reflect negligible group separation. **(B–D)** Volcano plots showing differentially expressed metabolites: **(B)** DX1 vs. DX4; **(C)** DX2 vs. DX4; **(D)** DX3 vs. DX4. Red dots = significantly upregulated metabolites, blue dots = significantly downregulated metabolites, grey dots = metabolites without statistical difference. **(E)** Heatmap tree of metabolite profiles under different treatments. Three biological replicates per group, with three technical replicates for each sample.

PCA (Figure 5A) displayed distinct separation of four groups, with PC1 and PC2 explaining 34.30 and 22.10% total variance (R = 0.6148, *p* < 0.001), and compact QC clusters validated data stability. To further discriminate group metabolic profiles, OPLS-DA and permutation tests confirmed reliable grouping without overfitting (Supplementary Figure 2). Metabolites with |Log₂FC| > 1 and *p* < 0.05 were defined as differential compounds: 11 upregulated and 3 downregulated metabolites in the dihydroquercetin group (Figure 5B), 3 upregulated and 3 downregulated metabolites in the ceftazidime group (Figure 5C), and the largest number of altered metabolites (13 upregulated, 12 downregulated) in the combination group (Figure 5D), reflecting stronger metabolic reprogramming upon combined treatment. Heatmap clustering (Figure 5E) illustrated suppressed pyocyanin, pyochelin, Sn-glycerol-3-phosphate and L-rhamnulose in treated groups, with the lowest levels in the combination, implying inhibited virulence and biofilm matrix synthesis; conversely, citric acid (a key TCA cycle intermediate) was lowest in the control, moderately increased under ceftazidime monotherapy, and peaked in the combination group. KEGG enrichment analysis of differential metabolites (DX3 vs. DX4) revealed three enriched pathways (Supplementary Figure 3): valine, leucine and isoleucine biosynthesis, pyruvate metabolism, and purine metabolism. The amino acid biosynthetic pathway displayed the highest rich factor and lowest *p* value, while central carbon and purine metabolism were also perturbed.

## 4 Discussion

Mucoid *P. aeruginosa.* is a primary pathogen driving chronic, recurrent human infections. *mucA* mutations trigger excessive alginate and extracellular polymeric substance secretion, which promotes dense biofilm formation and confers high-level antimicrobial resistance (Sautter et al., 2012; Ryall et al., 2014). Ceftazidime, a third-generation cephalosporin, inhibits penicillin-binding proteins to block cell wall synthesis and exert bactericidal activity (Shields et al., 2015); showing potent efficacy against planktonic non-mucoid *P. aeruginosa*. However, mature biofilms create a physical diffusion barrier that limits ceftazidime penetration, sharply reducing its activity against embedded sessile bacteria (Otani et al., 2018; Bowler et al., 2012). Given the limited therapeutic efficacy of single-antibiotic regimens against biofilm-mediated resistance, developing plant-derived adjuvants and synergistic combination therapies has become a critical research priority in anti-infective pharmacology.

Herein, we performed *in vitro* susceptibility testing on 41 clinical mPA isolates. Over 90% of strains displayed synergistic or additive antibacterial interactions when exposed to dihydroquercetin plus ceftazidime, confirming the promising therapeutic potential of this combination regimen. K-B disk diffusion assays indicated 87.8% of clinical isolates remained ceftazidime-susceptible, a finding inconsistent with previous resistance reports. Two key factors explain this discrepancy. First, most prior mechanistic studies relied on the laboratory reference strain PAO1 (Otani et al., 2018; Bowler et al., 2012), whereas our work exclusively employed clinical isolates with distinct physiological traits and intrinsic resistance backgrounds. Notably, wild-type laboratory-adapted PAO1 is inherently non-mucoid, while *mucA22* mutant PAO1 derivatives are frequently engineered and adopted for laboratory investigations of the mucoid phenotype, which creates an important phenotypic distinction from unmodified PAO1 that readers should bear in mind. Second, the K-B method only assesses planktonic bacteria and cannot recapitulate biofilm-linked resistance mediated by three-dimensional bacterial aggregates (Yin et al., 2019; Venkatesan et al., 2015). Additionally, PAO1 is inherently non-mucoid, with intrinsic resistance predominantly controlled by the *mexT*-regulated *MexEF*-OprN efflux pump (LoVullo and Schweizer, 2020). In contrast, the clinical mPA isolates tested herein develop resistance via *mucA*-driven alginate overproduction (Lemay-St-Denis et al., 2021), meaning planktonic susceptibility results cannot fully reflect antibiotic performance within biofilm microenvironments.

Plant-derived natural compounds such as dihydroquercetin represent attractive antibiotic adjuvants, as they rarely trigger bacterial resistance and exert multi-target pharmacological effects. Shevelev et al. (2020) reported that dihydroquercetin alone possesses weak bactericidal activity but enhances antibiotics (e.g., levomecol) by alleviating inflammation and remodeling the local immune microenvironment. After partial bacterial clearance by antibiotics, dihydroquercetin mitigates persistent pathogen-induced inflammation to prevent infection relapse and facilitate complete pathogen eradication. Consistent with these observations, our data demonstrated limited standalone activity of dihydroquercetin against mPA, yet co-treatment with ceftazidime generated robust synergistic or additive effects across most isolates (FICI ≤ 1.0). Time-kill curves further verified that monotherapy merely slows bacterial proliferation, whereas combined treatment achieves pronounced synergistic growth suppression.

Effective biofilm clearance is essential to resolve persistent mPA infections. Our data revealed that single drugs fail to eradicate mature biofilms efficiently, while combination therapy drastically reduces biofilm biomass. Mechanistic assays uncovered a dual synergistic mode of action. First, dihydroquercetin compromises membrane/biofilm integrity and increases permeability, enabling greater intracellular ceftazidime delivery to its molecular target and boosting bacterial susceptibility. Second, dihydroquercetin markedly suppresses transcription of core quorum-sensing regulator *lasR*, exopolysaccharide synthesis gene *pelA*, and flagellar motility gene *fliC*. These results corroborate the findings of Benin et al. (2023), who demonstrated that dihydroquercetin attenuates virulence and biofilm formation by interfering with the *Pseudomonas* quorum-sensing cascade, supporting its utility as an antibiotic adjuvant. This dual-action regimen establishes a multi-layered therapeutic approach combining membrane barrier disruption, virulence pathway inhibition, and targeted bactericidal activity, which efficiently targets sessile biofilm-embedded bacteria and outperforms monotherapy comprehensively.

Untargeted LC–MS metabolomics enables global profiling of bacterial physiological reprogramming under antibiotic stress. Far more metabolic alterations were detected in the combination group than in either monotherapy group, indicating that co-administration induces more extensive metabolic remodeling in mPA. Hierarchical heatmap clustering showed monotherapy moderately inhibits quorum-sensing-dependent virulence metabolites (pyocyanin, pyochelin) and biofilm matrix precursors (Sn-glycerol-3-phosphate, L-rhamnulose), with the strongest inhibitory effects observed upon combined treatment. Published work confirms pyocyanin and pyochelin biosynthesis is tightly governed by quorum sensing, and these metabolites act as core virulence factors governing biofilm maturation (Yang et al., 2020; Hirakawa et al., 2021). Sn-glycerol-3-phosphate (Liu Y. et al., 2022) and L-rhamnulose (Nickzad and Déziel, 2014) are likewise indispensable metabolites for biofilm assembly. Collectively, these results demonstrate that the combinatorial treatment suppresses bacterial virulence and blocks biofilm matrix biosynthesis at the metabolic level.

Citric acid, a key tricarboxylic acid (TCA) cycle intermediate, was significantly enriched in the combination group, representing a characteristic metabolic adaptation of mPA to drug pressure. Intact biofilm-resident bacteria in the control group exhibit low metabolic activity. Drug exposure, particularly dihydroquercetin, disrupts biofilm architecture and releases metabolically active planktonic cells, accelerating TCA cycle flux and citric acid accumulation. Cerca et al. (2014) showed that biofilm-detached planktonic bacteria undergo profound physiological remodeling with elevated metabolism and antibiotic susceptibility. Wan et al. (2018) further validated that biofilm-embedded cells display repressed metabolism, a phenotype central to biofilm-associated resistance. The negative correlation between citric acid levels and biofilm-related metabolites observed here further validates the potent biofilm-disrupting capacity of dihydroquercetin when paired with ceftazidime. KEGG enrichment further indicated that altered metabolites upon combination treatment were mainly enriched in valine, leucine and isoleucine biosynthesis, pyruvate metabolism and purine metabolism. Collectively, the drug combination may interfere with central carbon metabolism and amino acid homeostasis of mucoid *P. aeruginosa*, inhibit the synthesis of QS-dependent virulence factors, enhance antibacterial effects, and exert synergistic anti-biofilm and antibacterial activity.

This study has several inherent limitations. First, all functional assays were performed exclusively *in vitro*; no *in vivo* animal infection models or pharmacokinetic analyses were conducted, so the in vivo therapeutic efficacy and biosafety of this combination regimen remain unvalidated. Although three phenotypically distinct mPA isolates (susceptible, intermediate, resistant) were tested, testing on larger cohorts of clinical isolates is required to generalize the present findings to broader mPA populations. In addition, mucoid phenotype identification relied solely on macroscopic observation of colonial morphology; quantitative alginate/uronic acid colorimetric quantification was not performed to measure EPS secretion levels, which limits the availability of direct quantitative evidence supporting treatment-induced shifts in mucoidy. Second, our mechanistic investigation was restricted to transcriptomic and metabolomic profiling, with no proteomic validation of quorum-sensing and biofilm-associated proteins. We found dihydroquercetin combined with ceftazidime exerts greater antibacterial synergy than monotherapy. The combination enhances anti-mPA activity by impairing membrane and biofilm integrity, inhibiting quorum-sensing/biofilm gene expression and rewiring core bacterial metabolism. These preliminary mechanistic findings await further in-depth experimental validation. However, these mechanistic interpretations remain preliminary and require deeper experimental validation. Furthermore, our qRT-PCR assay only quantified expression of QS receptor and biofilm matrix genes, omitting upstream QS synthase genes (*lasI, rhlI*) and PQS pathway marker genes (*pqsA, mvfR*), which prevents full reconstruction of the complete QS regulatory signaling network. Finally, the optimal concentration ratio for the two therapeutic agents was not determined in the current study.

Future work will establish mPA pulmonary and wound infection animal models to systematically evaluate the *in vivo* therapeutic efficacy, systemic toxicity and overall biosafety of the dihydroquercetin–ceftazidime combination. We will also perform colorimetric uronic acid quantification to measure alginate production and generate quantitative data describing how the drug combination suppresses synthesis of mucoid extracellular polysaccharides. Multi-omics analytical frameworks integrating transcriptomics, proteomics and qRT-PCR will be applied to fully dissect the underlying molecular regulatory mechanisms. Specifically, follow-up multi-gene qRT-PCR assays covering the complete quorum-sensing signaling cascade will be conducted to systematically clarify the pathway through which the combination inhibits quorum sensing. In addition, subsequent experiments will screen the optimal matching ratio of the two agents and optimize delivery formulations for dihydroquercetin to improve its aqueous solubility and bioavailability, ultimately overcoming translational barriers and facilitating clinical translation of this synergistic anti-biofilm therapeutic regimen.

## 5 Conclusion

This study demonstrates that dihydroquercetin potentiates ceftazidime against mPA. It enhances bacterial biofilms/membrane permeability to facilitate antibiotic action, and co-treatment jointly suppresses the transcription of quorum-sensing and biofilm-associated genes. The combination triggers extensive metabolic reprogramming by perturbing central carbon, amino acid and purine metabolism, reducing virulence metabolites and biofilm matrix precursors to produce robust synergistic antibacterial and anti-biofilm activity.

## Data Availability

The datasets presented in this study can be found in online repositories. The raw data supporting the conclusions of this article are available in Zenodo, https://doi.org/10.5281/zenodo.22082949.
