# Supplementary material for: Mechanisms of synergistic antibacterial and anti-biofilm effects of dihydroquercetin combined with ceftazidime against mucoid pseudomonas aeruginosa
Source: Front Microbiol. 2026 Sep 9;17:1945324. doi: 10.3389/fmicb.2026.1945324 (PMC13597665; doi:10.3389/fmicb.2026.1945324)
Supplement: Supplementary file 1 [file Supplementary_file_1.DOCX]

Supplementary Material

## Supplementary Table

**Supplementary Table 1** Basic clinical information of the 41 mPA isolates.

| **Variable** | **strains（n=41）** |
| --- | --- |
| **Age（years）** | 64(58~ 69） |
| **Gender [n(%)]** |  |
| Male | 13(31.7%) |
| Female | 28(68.3%) |
| Specimen source**[n(%)]** |  |
| sputum and throat swabs | 32(78.0%) |
| bronchoalveolar lavage fluid(BALF) | 7(17.2%) |
| urine | 1(2.4%) |
| blood | 1(2.4%) |
| **Department[n(%)]** |  |
| Respiratory Medicine Ward | 28(68.2%) |
| Infection Ward | 2(4.9%) |
| Thoracic Surgery Ward | 2(4.9%) |
| Outpatient Clinic | 2(4.9%) |
| other departments | 7(17.1%) |
| **Bronchopulmonary infection[n(%)]** |  |
| Yes | 38(92.7%) |
| No | 3(7.3%) |
| **Tumor[n(%)]** |  |
| Yes | 5(12.2%) |
| No | 36(87.8%) |

## Supplementary Figures

**Supplementary Figure 1.** Cumulative rate curves of Dihydroquercetin (A) and Ceftazidime (B) monotherapy and combination therapy.41 isolates were examined, and three technical replicates were set for each sample.

**Supplementary Figure 2.** OPLS-DA analysis of metabolomic profiles between treatment groups(DX1-DX3) and blank control (DX4). OPLS-DA score plots for DX1 vs DX4 (A), DX2 vs DX4 (C), and DX3 vs DX4 (E), respectively. Component 1 represents the predictive principal component, and Orthogonal Component 1 denotes the orthogonal principal component; the percentage values indicate the proportion of explained variance. Corresponding permutation test histograms (200 permutations) of the OPLS-DA models for DX1 vs DX4 (B), DX2 vs DX4 (D) and DX3 vs DX4 (F).Three biological replicates per group, with three technical replicates for each sample.

**Supplementary Figure 3.** KEGG enrichment analysis of differential metabolites between DX3 and DX4. The horizontal axis represents the rich factor, the vertical axis represents enriched pathways. Dot color corresponds to P-value, and dot size indicates the number of enriched differential metabolites.Three biological replicates per group, with three technical replicates for each sample.
